# Achieving Low Latency Communications in Smart Industrial Networks with Programmable Data Planes

**DOI:** 10.3390/s21155199

**Published:** 2021-07-31

**Authors:** Asier Atutxa, David Franco, Jorge Sasiain, Jasone Astorga, Eduardo Jacob

**Affiliations:** Department of Communications Engineering, Faculty of Engineering, University of the Basque Country UPV/EHU, Alda. Urquijo S/N, 48013 Bilbao, Spain; david.franco@ehu.eus (D.F.); jorge.sasiain@ehu.eus (J.S.); jasone.astorga@ehu.eus (J.A.); Eduardo.Jacob@ehu.eus (E.J.)

**Keywords:** P4, data plane programming, IoT, industrial communications, time-critical

## Abstract

Industrial networks are introducing Internet of Things (IoT) technologies in their manufacturing processes in order to enhance existing methods and obtain smarter, greener and more effective processes. Global predictions forecast a massive widespread of IoT technology in industrial sectors in the near future. However, these innovations face several challenges, such as achieving short response times in case of time-critical applications. Concepts like in-network computing or edge computing can provide adequate communication quality for these industrial environments, and data plane programming has been proved as a useful mechanism for their implementation. Specifically, P4 language is used for the definition of the behavior of programmable switches and network elements. This paper presents a solution for industrial IoT (IIoT) network communications to reduce response times using in-network computing through data plane programming and P4. Our solution processes Message Queuing Telemetry Transport (MQTT) packets sent by a sensor in the data plane and generates an alarm in case of exceeding a threshold in the measured value. The implementation has been tested in an experimental facility, using a Netronome SmartNIC as a P4 programmable network device. Response times are reduced by 74% while processing, and delay introduced by the P4 network processing is insignificant.

## 1. Introduction

In the last years, small sensors have proliferated along with Internet of Things (IoT) networks and communication protocols to massively gather any kind of data and transmit it in real-time. According to the GSM Association, the number of IoT devices will grow to 25.1 billion by 2025 [1]. Additionally, Ericsson forecasts that there will be over 30 billion connected devices by the end of 2025 [2]. Thanks to IoT networks and devices, any kind of data, from vital signs to manufacturing processes, could be pervasively monitored. This massive data gathering, supported by artificial intelligence or machine learning technologies, allows creating smarter processes, improving decision making and response time.

In the specific context of industry and advanced manufacturing, massive data gathering enabled by IoT devices and networks could be used to improve the efficiency of manufacturing processes, enhance predictive maintenance and, overall, save costs. For example, collected data could be fed into machine learning algorithms specifically designed to predict failures or manufacturing defects, allowing in this way to avoid these issues before they occur. In this context, the most widely considered implementation proposals present a design where algorithms and process enhancing applications are deployed in centralized data centers, requiring constant communications between the cloud and the factory equipment. Therefore, data center servers can remotely manage multiple factories from the cloud, strengthening the scenario for future industrial communications.

However, for the above mentioned scenarios to achieve the expected success, near real-time communications must be guaranteed between the factory equipment and the cloud. Nevertheless, in the case of applications and services involving industrial IoT (IIoT) devices, achieving low latencies remains an unresolved challenge. On the one hand, the high amount of data transmitted by massively deployed IIoT devices could cause congestion in the IIoT network. On the other hand, long distances from the devices to the centralized server can also cause important delays in packet transmission. All of these issues hinder the most demanding objectives of achieving very fast communications in next generation networks and the success of novel IIoT-based Industry 4.0 applications.

In this regard, concepts such as in-network computing (INC) [3] or edge computing have been developed to improve response times in applications with strict timing constraints. The main idea behind these concepts is to bring services and data processing closer to the producer and consumer devices, frequently to the edge of the network.

In addition to this, data plane programming (DPP) has become an excellent method for flexible network computing execution, providing the opportunity to implement network functions into the switches and other network devices. In this context, P4 [4] is a data plane programming language that allows packet definition and processing in a high abstraction level. Therefore, DPP through P4 language offers a suitable environment for service deployments and packet processing closer to the end user in a network.

The research question this paper addresses is how to improve the efficiency of communications in industrial processes in two main ways: (1) reducing the communication delay and (2) avoiding unnecessary traffic. The solution is to bring the processing closer to data generating devices, which are at the edge of the network. To achieve this, the DPP method is analyzed in-depth. In this context, this paper presents the concept and the implementation of an in-network processing system for IIoT communications, in order to reduce latency of data transmissions and improve response times in the bidirectional link between the end IIoT devices and the data processing servers. This enhancement of performance parameters is crucial for the feasibility of modern applications in the industrial sector, as it is necessary to develop communication and connectivity alongside fabrication processes to make progress.

More specifically, the targeted scenario consists of a network of IIoT devices that gather data about a manufacturing process and transfer it to a cloud server for its storage and processing. For data transfer, the Message Queuing Telemetry Transport (MQTT) industrial communication protocol is used and the data processing consists of comparing the value conveyed in the MQTT message with a threshold, and if it exceeds the threshold, generating an alarm message. In such a scenario, the main idea of the proposed INC application is to process the data carried by MQTT [5] packets at the edge of the IIoT network, replacing the cloud data processing. That is, the INC application reads the value inside the MQTT message, compares it with a predefined threshold and performs a different action depending on whether the value is below or above the threshold. Specifically, if the value carried within the MQTT message exceeds a predefined threshold, the message is automatically forwarded to an edge broker for faster processing, while a copy of the message is sent to the cloud broker for its storage. In this way, packets with values exceeding the threshold are processed faster than in the traditional paradigm, while it is still possible to keep the control and management of IIoT devices in the centralized broker in the cloud. If the value is below the threshold, the packet is just forwarded to the cloud broker.

Taking this into account, the main objective of this paper is to present the design of an IIoT communication system that leverages INC and programmable data planes to reduce response times of time constrained applications. Therefore, the main contributions of this paper can be summarized as follows:

- Definition of a communication architecture, including the programmable data plane design, in the context of an IIoT scenario.
- Inclusion of application processing inside the network for time constrained IIoT services.
- Showcase implementation of a solution for response time reduction in IIoT environments.
- Comprehensive performance evaluation of the proposed architecture in order to prove its effectiveness for the targeted scenarios.

The rest of the paper is organized as follows: Section 2 reviews the concepts and technologies analyzed in this paper. Section 3 surveys the related work. Then, Section 4 describes the proposed system, and Section 5 describes the implementation and the design of the performance tests. Section 6 shows the obtained results, and finally, Section 7 summarizes the main conclusions and future research directions.

## 2. Concepts and Technologies

The industry is renewing itself, and telecommunications have more relevance and impact than ever in production processes. Intelligent devices have increased in number in factories, including sensors able to measure any physical parameter, such as temperature, acceleration, and humidity. All the aforementioned data can be used to improve processes and optimize the performance of machinery in industrial environments. However, in order to achieve this objective, multiple important points such as data processing, transmission and storage must be taken into account. The introduction of intelligence in industrial processes can only be beneficial if solutions are proposed for these challenges.

This section presents and describes the most important concepts and technologies involved in the INC system proposed in this paper. First, IIoT communications are analyzed; then the architecture of industrial networks is explained, including different paradigms that can be used; and finally, the P4 programming language for networking devices is introduced.

### 2.1. IIoT Communications

Industrial communications have multiple factors that affect the quality and efficiency of services and applications. Some of those are straightforward elements, such as hardware equipment or physical technologies used for transmission (electrical or optical). However, there are other less-obvious aspects, such as the application layer protocol, which has a substantial impact on the performance of the communication, mainly due to the structure of IoT packets. These packets do not carry abundant information, because sensor measurements commonly have few bytes, which results in a poor header-to-payload rate. This means that sending a packet requires attaching a lot of control information as packet headers (Layer 2, IP, TCP…), just to send a small value over all the layers. IIoT specific application layer protocols provide a solution for that problem, having other benefits for sensors such as low resource usage.

There are multiple specific protocols for IIoT message transmission in industrial scenarios, such as MQTT [5], XMPP [6] and OPC UA [7], which rely on a publisher–subscriber model, or Modbus [8] and CoAP [9], which follow a request–response model. Apart from the communication model, there are additional differences among these protocols, such as the used transport layer protocol (TCP, UDP, SCTP…), the implemented security mechanisms (TLS, X.509 certificates…), or the use of intermediary entities, such as message brokers.

MQTT is one of the most common protocols for IIoT communications, following a publisher–subscriber model consisting of three different entities: the publisher, the subscriber, and the intermediate broker. This broker acts as an intermediary between the other two endpoint-communicating entities, as the publisher sends messages to a topic in the broker and any subscriber can join a topic to receive the messages that are published on it. In an IIoT scenario, a topic can represent a measurement, such as temperature, acceleration, etc.

**Figure 1:**
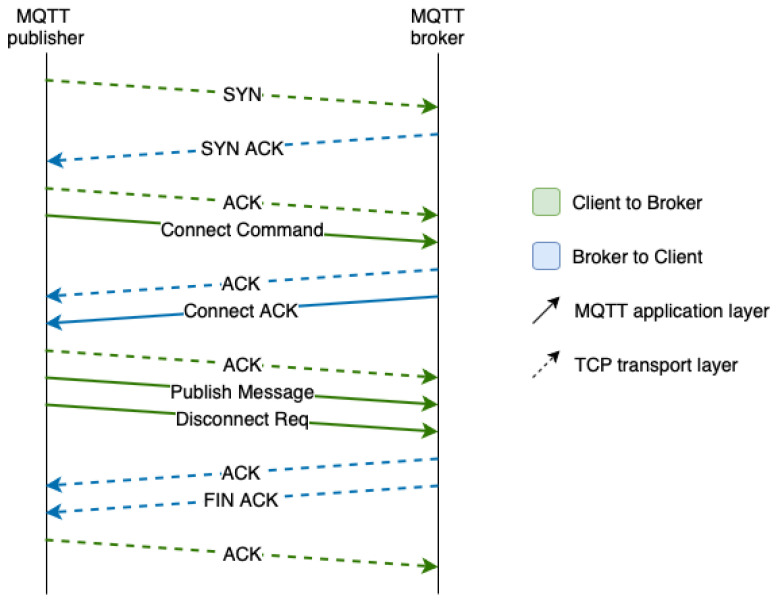
MQTT protocol messages for a MQTT publication.

MQTT is sent over TCP, a lossless and bidirectional transport layer protocol. This means that a connection is established between a client and a server, keeping track of the session, which requires the exchange of additional messages. In fact, Figure 1 shows that before sending a publication, the publisher has to establish a TCP connection with the broker server through the TCP 3-way-handshake, then establish the application layer connection, send the message with the data, and finally close the connection.

### 2.2. Data Processing in Industrial Networks

**Figure 2:**
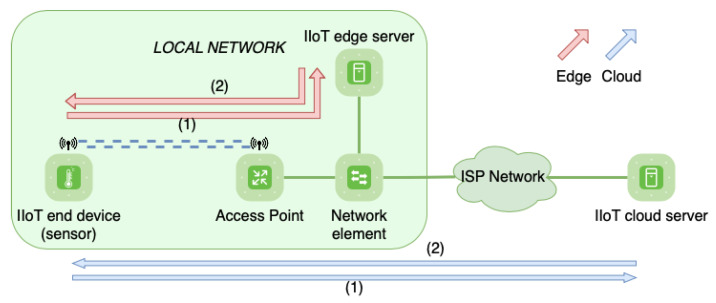
Edge and cloud communication in an industrial network.

As mentioned in the previous subsection, features like communication protocols have a deep influence on performance. Thus, optimizing them is a crucial matter. There are plenty of methods and technologies available to improve communication parameters, such as network routing protocols, processing algorithms, or virtualization of resources. In this context, one of the most innovative concepts and with the most projection is in-network computing (INC), along with edge and cloud computing paradigms. INC extends the concept of edge computing by leveraging the data plane of networking devices to implement packet processing functions at line rate. This means that devices that traditionally have only provided forwarding of packets can now implement some intelligence to process the information transmitted in those packets and make decisions based on that information. Edge and cloud are different parts of a network that are distinguished by their locations, as the first one is closer to the end devices and the second one is usually in a remote station. These sites contain computing and storage servers, usually having more resources in the cloud than in the edge. Different locations make them suitable for different objectives. Edge servers offer superior results when it comes to response times from end devices, being adequate for services with strict timing constraints. Cloud servers, however, are usually gathered in data centers, centralizing network control in a single point. This simplifies network organization and reduces resource expenses, as having centralized equipment and servers is more efficient than duplicating every service in each local network. Figure 2 shows the communication in both cases, edge (red) and cloud (blue).

INC offers numerous use cases in the industry. A clear example is the control of packet routing, which consists of processing packets or packet flows inside network elements (like switches) and taking decisions about how to route them without the intervention of a centralized controller. Filters can also be applied, which can be used for packet routing (as in the previous case) or even for detecting inconsistencies, such as duplicated packets or network attacks. Another interesting use case is data preprocessing; that is, the deeper inspection of packets in order to read higher level information before the packet reaches the final server. This can be really useful, as it can be combined with previous routing alternatives to process packets before they arrive at the destination, in this way providing a faster response.

### 2.3. DPP and P4

The implementation of INC requires the deployment of processing functions in the network elements. Normally, these elements, such as switches, may have the ability to apply some fixed functions defined by the manufacturer. However, there are some programmable switches that incorporate the possibility to design custom data planes, which makes it possible to describe and deploy specific functions. Therefore, DPP and programmable switches are the ideal approaches for the implementation of INC and, in this case, the processing of industrial communications. In this regard, P4 language is used to define the behavior of programmable switches to process packets in the network. P4_16_ is the latest version, and it has been an extensively investigated topic in the last years [10]. It supports multiple targets such as ASICs, NPUs, software switches or FPGAs, and also supports multiple architectures. These architectures can be defined as programming models that are logically located between the P4 program and the target, so that a P4 program is written for a specific architecture, and all targets that support that architecture can implement the program.

**Figure 3:**
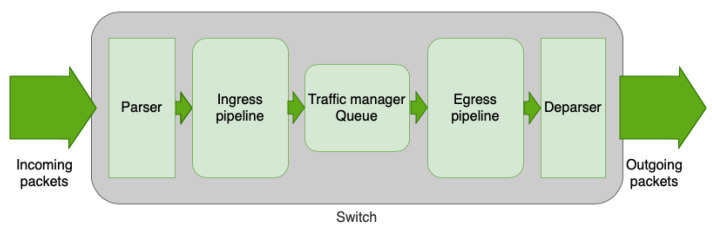
Architecture of a P4 switch.

P4 programs describe the packet processing method that the target (switch) gives to the incoming packets. That processing is executed through a specific architecture, which is the internal composition of the target. It usually has five main phases: parser, ingress pipeline, traffic manager (queue), egress pipeline, and deparser. Figure 3 shows the graphical representation of the architecture, explained below.

- Parser: This is the first phase, and it deals with the processing of the packet to extract the headers and the different parts through a customized procedure. Headers are defined at the start of the code, being able to define specific fields for each one to later process the data inside them from received packets.
- Ingress pipeline: This is the phase for the logic of the switch, where fields are analyzed and actions are executed as a result of the obtained values (forward to a port, change the destination address, drop the packet…).
- Traffic manager queue: This phase is executed after the processing of packets in the ingress pipeline, and it queues packets to their exit.
- Egress pipeline: This is a stage similar to the ingress pipeline, in which it is possible to apply logic and execute actions just before sending the packet out of the pipeline.
- Deparser: This stage rebuilds the packet header by header, and finally, after this phase, the packet is sent through the corresponding port.

This programmable and flexible architecture allows for the implementation of INC functions by modifying the P4 program. More specifically, payload processing can be performed thanks to the parsing of all headers and fields. The targets in which the P4 program is deployed are able to read application layer information, and in the case of IIoT communications, MQTT protocol data can be processed. Thus, it is possible to carry out any action that could be executed in a data processing server. INC can be used to offload applications from a server to networking devices, answer directly to the endpoint device, or attach telemetry data to the packet. This processing can be implemented in any kind of scenario, as it can fully adapt to each specific deployment by adjusting the P4 program to process the required information.

## 3. Related Work

Data plane programming is a concept that has been studied substantially in the last years, as it provides crucial tools to enable all of the aforementioned use cases. Especially, P4 has been greatly studied to process packets in communication networks with the goal of enhancing their performance. On the one hand, [11,12,13,14] researched the measurement of network statistics using data plane programming with P4 language. Ref. [11] developed a congestion management mechanism that monitors the user activity and accordingly prevents users from monopolizing the network. Authors in [12,14] proposed a solution in multiple platforms (P4, FPGA, GPU, CPU, multi-core CPU, and OVS) for network measurement reports in real time, based on elastic (instead of static) sketches in order to dynamically allocate memory for elephant flows. Authors in [13] proposed a data gathering method and a monitoring system for network management that does not use the control plane, implementing the entire solution in the data plane.

On the other hand, ref. [15,16,17,18,19] are examples of papers that studied the specific detection of huge flows called “heavy hitters”, which entail exposure to congestion of networks. These works investigate the anticipation and early detection of those flows in order to answer with actions that boost the performance of data transmissions. Ref. [15] presented a fast and compact invertible sketch that supports heavy flow detection with small memory allocation. Ref. [16] explained a solution to incrementally deploy programmable devices in a legacy network to be able to monitor the network in an optimal way and detect heavy hitters with a high chance. Ref. [18] presented a solution that is an accurate and efficient mechanism for collecting and analyzing packets when traffic is complex. Ref. [19] is a paper that presented a network-wide heavy hitter detector implemented in one-big-switch.

Additionally, there is a large record of industrial works related to IoT, network data processing and P4, because sensor usage is one of the main pillars of innovation in the industry. Refs. [20,21,22,23] presented solutions of gateways implemented in P4. Ref. [22] proposed a multi-protocol edge-based architecture for wide IoT networks using P4 switches between devices and including an SDN controller. Another application that has been analyzed in many articles is the aggregation and disaggregation of IoT packets with P4. This has been addressed in [24,25,26], and results in better efficiency of information transmission due to a better header-to-payload ratio in IoT packets.

Two interesting papers in this field are [27,28]. Authors in [27] proposed the offloading of latency-critical services into the network using programmable data planes in order to improve the performance of industrial environments, more specifically robotics. Ref. [28] is also based on industrial networks, and presented a solution for offloading monitoring and measurement applications to the network elements using programmable data planes with P4 language. However, on the one hand, the solution of [27] is to create a static response message that is answered directly to the robot, so it does not have the possibility to further process the information in a dedicated server. On the other hand, ref. [28] involves the control plane through SDN and industrial controllers for the population of rule tables in the switch, which in addition to the use of registers, makes the deployment more complex. Furthermore, these two works did not develop some essential aspects in the implementation of industrial technologies. The mentioned studies are theoretical, proving their solutions in virtual, simulated, or emulated environments (with bmv2 software switch), that do not provide a complete capability of gathering meaningful results.

Our paper proposes a solution to those problems, presenting a hardware implementation of the IIoT environment. This is an innovative approach, mainly due to the current lack of real implementations of INC in industrial environments using state of the art DPP. Simulations and mathematical analysis are well-known methods to test and validate proposals, but our scope is beyond theoretical investigation. In fact, the aforementioned simulations and mathematical analysis are an abstraction of reality, so taking all factors into account becomes virtually impossible. Therefore, our testbed environment is built with physical equipment, which allows us to test network processing concepts and the decision making in the data plane, aiming for the enhancement of performance in communications. Additionally, we propose an efficient solution implemented entirely on the data plane that establishes a well-balanced task delegation between the network and data center servers.

## 4. Design of the INC-Based System

This paper presents an INC-based system focused on improving the processing of industrial communications, achieving shorter response times and reducing the amount of transmitted data. More specifically, the solution presented in this paper consists of processing a sensor measurement publication message (aforementioned MQTT publish message), analyze it in the programmable switch, and if it exceeds a threshold, duplicate and send it through another port towards the edge broker, calling that new duplicated message an “alarm”. The transmission to an edge broker results in a lower response time compared with the cloud communication. Besides, the transmission between the switch and the edge broker is much less congested than the main link to the cloud data center, which usually entails going through a public network, thus reducing link failure possibility and avoiding network congestion problems that would slow down TCP transmissions.

### 4.1. Proposed Architecture

Industrial networks rely both on cloud and edge computing approaches, each one being optimal for different goals. These two approaches can coexist in an industrial network, but frequently this requires full duplication of all transmitted packets and duplicated processing in both end-sites. However, we designed a distinct paradigm for IIoT scenarios, which takes advantage of the strengths of each solution depending on the exact communicated packet. In other words, our architecture manages each message depending on the purpose of that transmission. In this way, a “hybrid” operation mode is implemented, improving response times for time constrained communications, while keeping track of all IIoT data in a centralized site.

**Figure 4:**
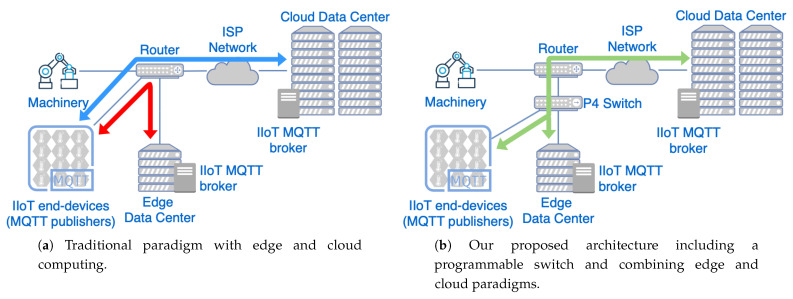
Comparison of edge/cloud computing architectures against our proposed solution including the programmable network element. (**a**) Edge/cloud computing architecture. (**b**) Proposed solution.

Figure 4a shows the communication with the traditional cloud and edge computing, while Figure 4b shows our design including the programmable network device. The main difference between both network topologies is the introduction of a programmable switch. This network device processes the information transmitted through the network and decides the appropriate forwarding that should be given to each packet, implemented by the INC application that is explained in the next section.

### 4.2. Design of the Data Plane

As mentioned before, P4 allows us to describe the processing of a packet in a programmable switch. In this case, the switch first needs to process the whole packet and read all fields in each header, and then detect if the value is over a threshold in the MQTT payload. Accordingly, when an MQTT message carries such a value, the switch has to generate an alarm (clone the packet) and send it to the edge broker for faster processing. All this process has to be defined in all the aforementioned stages of the architecture.

**Figure 5:**
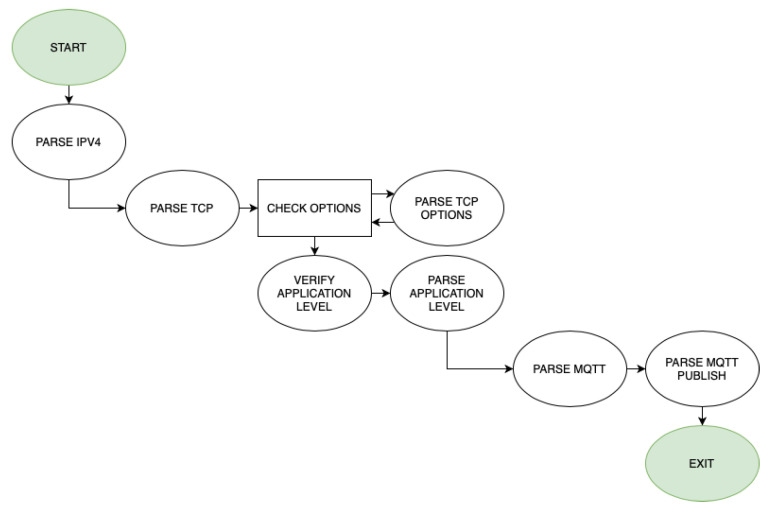
Flux diagram for the parser phase.

When it comes to the parser, P4 supports the processing of headers without layer limitation, being able to extract application layer headers. The main problem is processing headers with optional parts, as is the case with TCP. TCP has a mandatory part of 20 bytes, and can have one or multiple optional headers of different sizes after that (so it has a variable length). This means that it is not possible to define headers statically in the P4 program, neither to define a simple and linear algorithm for header extraction. However, P4 has variable length field definitions (varbit), which are parsed by keeping track of the remaining IP and TCP header lengths. The flux diagram for the parsing phase can be seen in Figure 5.

**Figure 6:**
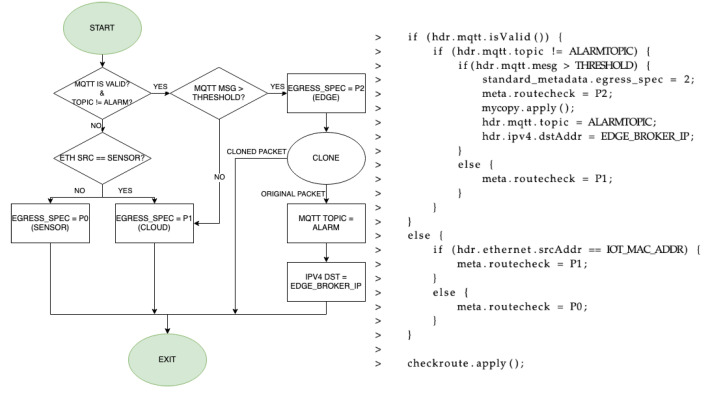
Flux diagram and code for the ingress phase.

The network processing of MQTT packets is done in the logic of the switch (ingress and egress). In the ingress pipeline, which is shown in Figure 6 (flux diagram and code), the first thing is to analyze if the received packet has a valid MQTT header and to check which is the topic that is being published. Two topics are used in this case, “test” and “alarm”. Publish messages with “test” topic are analyzed in the P4 switch, while generated alarm packets will carry an “alarm” topic. Therefore, depending on the topic of the packet received in the switch, different processing is done for the data. If the packet does not have an “alarm” topic, it means that the packet must be processed, which in this particular use case is checking if a threshold is exceeded. If that is the case, an alarm is generated. This is done by cloning the packet in order to get an exact copy of it, and then modifying values of various header fields in the original packet to change the destination and the message it carries. Particularly, it is necessary to change the output port of the programmable switch (P2, edge), IP destination address and the MQTT topic, although it is optional to modify other parameters such as the destination port of the transport layer, the MQTT identifier, or any parameter of the Ethernet header. If it is not a MQTT packet, it means that the packet should not be processed by the data plane because it is not an IoT message. In that case, the packet should go through the programmable switch in a transparent way, from one of the ports to the other, forwarding it to the next hop. To do so, the sender MAC address is analyzed to check if it was received from the IIoT device (first port, P0) or the other side of the network (second port P1, server side). Depending on the source, the packet is forwarded to the other physical port in the programmable switch in order to maintain its original transmission path.

**Figure 7:**
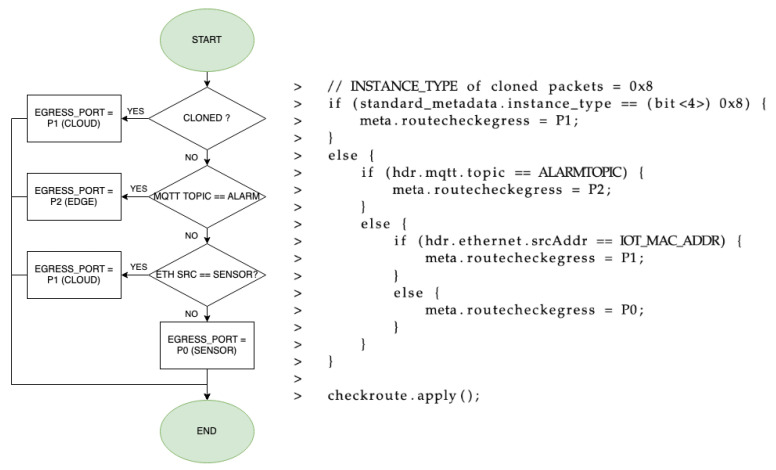
Flux diagram and code for the egress phase.

The egress phase, presented in Figure 7 (flux diagram and code), checks if the packet is a clone (generated in the ingress) or if it is sent by the IIoT device. Accordingly, the corresponding output port is set for that packet using the *egress_port* metadata field. If it is a clone, it means that the packet is an unmodified copy of a threshold-exceeding MQTT publish message, so it is forwarded towards the cloud MQTT broker (P1). If it is not a clone, the topic is analyzed in order to find the threshold-exceeding MQTT publish message related to the previous clone. In that case, it is sent to the edge (P2). If not, the origin of the packet is checked as in the ingress pipeline, so if it comes from one of the brokers, it is sent to the IIoT device (P0). However, if it comes from that IIoT device, it is sent to the cloud broker (P1).

Finally, before rebuilding the packet in the deparser, it is mandatory to update checksum values in the headers that had fields modified, such as IP and TCP. To do so, an extern is used, which is a type of action or function that is specific to the architecture. Specifically, the extern used is *Checksum16*, which receives input parameters that are necessary for the checksum value calculation and updates it in the corresponding packet. This is crucial, because if the IP address and MQTT topic are modified in a packet and the checksum is not updated, that packet would be discarded upon receiving it in the destination due to an incorrect checksum value.

## 5. Implementation and Performance Evaluation

This section explains the performance evaluation carried out to show the suitability of the proposed solution, including the implementation of a testbed of the targeted network and the design of the validation tests.

### 5.1. Testbed Architecture

The testbed used to validate the proposed INC system uses real equipment and a hardware-based P4 target that implements the aforementioned data plane architecture. The validation is carried out on top of Smart Networks for Industry (SN4I) [29], a Network Function Virtualization- (NFV) and Software Defined Networking (SDN)-enabled experimental facility deployed across the Faculty of Engineering in Bilbao (EIB) and the Aeronautics Advanced Manufacturing Center (CFAA). The CFAA was launched by the University of the Basque Country along with a wide range of companies involved in manufacturing and Industry 4.0, with the aim of raising industrial competitiveness of the region through the use of novel machinery and manufacturing techniques. These two locations are interconnected through a Layer-2 SDN network at a rate of 10 Gbps. Within the EIB site network, the P4 switch is implemented using a Netronome SmartNIC, which has 2 physical 10 Gbps ports and the ability to create 128 virtual ports. Readers that would like to know more about the SmartNIC can refer to [30].

Both sites contain data centers that are equipped with the hardware and software components that enable the deployment of virtual services in the infrastructure, leveraging NFV technology. The complete infrastructure comprises several SDN switches and servers, amounting to a total computing and storage capacity of around 250 dual-threaded cores, 1 TB of RAM, and 25 TB of storage. Two OpenStack nodes deployed in EIB and the CFAA manage the resources of their respective locations, and three ONOS SDN controllers manage, respectively, the data plane connectivity in the EIB domain, the CFAA domain, and the Wide Area Network (WAN) between both locations. In other words, the role of SDN in SN4I is to reactively install the forwarding rules that enable the connectivity between VMs and physical equipment connected to either side of the infrastructure. Finally, an Open Source MANO (OSM) orchestrates the whole NFV infrastructure, exposed by the two OpenStack nodes, and manages the SDN controllers to control the routing.

In this context, the CFAA contains the IIoT devices and machinery that are considered to be located at the edge of the network, in the factory itself. For the validation tests in this paper, we have simulated this equipment in a virtual machine with 4 GB of RAM and 2 cores in the OpenStack that is located in the CFAA (factory). The EIB, in turn, includes the edge MQTT broker and the connection point with the ISP that provides access to the remote MQTT broker, which in our scenario is considered the cloud server.

### 5.2. Experiments and Measurements

**Figure 8:**
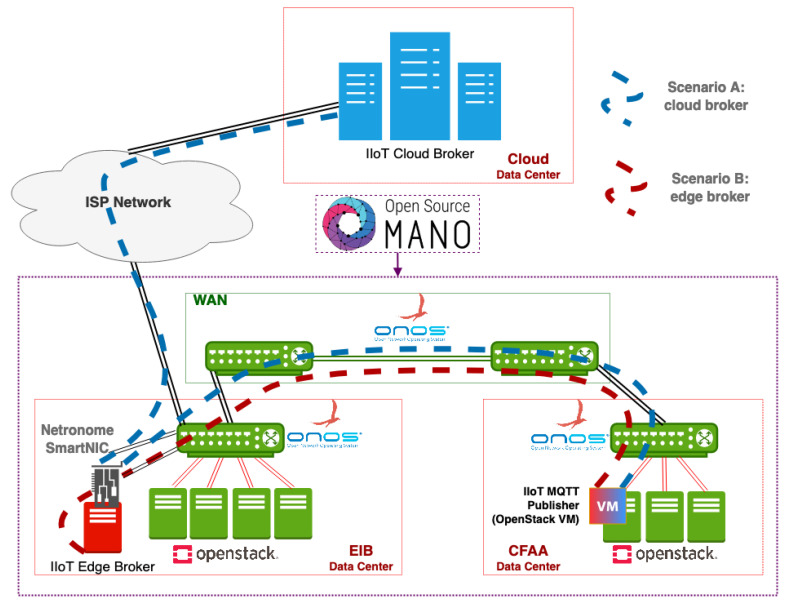
Testbed network of the industrial architecture based on SN4I.

This section explains the experiments that have been designed and performed in order to validate the solution presented in this paper. The experiments have been executed in two different scenarios of IIoT communication presented bellow and in Figure 8:

- Scenario A: This scenario comprehends the transmission of a message from the IIoT device to the SmartNIC, then to the cloud broker, and then the same path for the response.
- Scenario B: This scenario comprehends the transmission of a message from the IIoT device to the SmartNIC, then to the edge broker, and then the same path for the response.

The operation of the experiments is the following. Messages carrying temperature measurements are sent from the MQTT publisher to the cloud MQTT broker using the Mosquitto Linux package, which is an MQTT client/broker implementation. Those messages are analyzed upon arrival at the P4 SmartNIC, which decides where to send them. In the first scenario, where they are sent towards the cloud broker, this server processes them using a Python-based application to decide if the value exceeds a threshold. If that is the case, a response is sent to the publisher. However, in the second scenario where MQTT messages are sent to the edge broker, this server receives them with a Python application based on Scapy library, which automatically generates the response to the publisher. Response packets in both cases could be any kind of message, such as action triggering messages for industrial machines. For the sake of simplicity, we have decided to answer with MQTT alarm messages too, by subscribing the same IIoT device that publishes the measurements to the “alarm” topic in those brokers.

Key performance indicators (KPIs) have been defined for the performance evaluation of the solution through several experiments, basing the election on the environment of the solution and its objectives. Experiments have been repeated 1000 times to obtain statistically meaningful results and not to be linked to temporary or occasional conditions.

#### 5.2.1. Experiment 1: Response Time

The objective of the solution is to reduce the IIoT communication response time, which is one of the most crucial measurement indicators. This comprehends the transmission of the sensor measurement value to the destination broker, along with the transmission of the corresponding notification to the machinery. Two tests are performed inside this experiment, one for scenario A **(test 1.1)** and another one for scenario B **(test 1.2)**.

For the correct measurement, we have decided to take timestamps of the transmission in the same device, specifically at MQTT publishing and at response arrival. This solves time synchronization issues between distinct devices, because using the same device results in using a single clock as a point of reference. Time measurements have been taken with a precision of microseconds in order to achieve high accuracy.

#### 5.2.2. Experiment 2: Network Processing Delay

To analyze the effectiveness of the solution with SmartNIC processing inside the network, this experiment is aimed at measuring the communication delay introduced by the P4 processing at the SmartNIC. For this goal, four different tests have been conducted, and the corresponding delays measured. In the first **test (2.1)**, the traffic from the MQTT publisher travels to the cloud broker through the SmartNIC, where the corresponding INC is performed. In the second **test (2.2)**, the SmartNIC is removed and the traffic travels directly to the cloud broker, without any additional processing. As the delay in the communication with the cloud broker is higher than with the edge broker, we have decided to perform the third and fourth tests **(2.3 and 2.4)** measuring the same delays as in the first and second tests, but in the communications with the edge broker, both with and without the SmartNIC processing. In fact, as end-to-end delays in the IIoT-edge broker communications will be smaller than in the corresponding communications with the cloud, it will be easier to appreciate little variations in these delays. The aim of this experiment is to verify that processing in the SmartNIC does not introduce a delay big enough to counter the benefits exposed in the previous experiment.

#### 5.2.3. Experiment 3: Throughput Comparison between Edge and Cloud Communications

Another performance indicator is the throughput, which is critical as far as the expansion of massive IIoT deployments in factories is concerned. In fact, a single IIoT device cannot consume enough resources to be noticeable in current networks, but scaling this usage to thousands of devices could cause problems, especially when the traffic of other applications, such as video surveillance, are mixed in the same network. In fact, a single IIoT device could generate about 1.25 Kbps of data, as this general research article shows [31]. Thus, one thousand sensors would generate 1.25 MB/s, which is 10 Mbps of data rate just with MQTT communication. Furthermore, this transmission would consist of many small packets, which could be challenging to operate in a single broker because it is more demanding to process many small packets than fewer (but bigger) packets of the same total length.

Therefore, in this experiment, we concentrate on the throughput generated by the aforementioned alarm generation system. As previously explained, this processing can be performed both at the SmartNIC or at the cloud broker, and the aim of this experiment is to compare the throughput generated in these two cases.

Several tests have been performed with multiple percentages of original MQTT publish messages exceeding the threshold value, in order to analyze several possible cases: 1% (**test 3.1)**, 5% (**test 3.2**), 10% (**test 3.3**), 15% (**test 3.4**), 20% (**test 3.5**), 30% (**test 3.6**), and 50% (**test 3.7**). Among these tests, those with lower probabilities have more relevance in a real-world environment, as it is not a common situation to exceed the threshold of an IIoT measured parameter. However, in order to have a broader set of results to analyze, this experiment shows the results for up to 50% of packets above the threshold. It is reasonable to expect an almost linear increment in the throughput with an incremental probability of exceeding the threshold value, as this probability is directly proportional to the number of alarm packets generated, and therefore to the throughput. However, edge communication will be much lower than the cloud communication due to the absence of TCP connection establishment messages in the first one, thanks to the SmartNIC filtering of only publication messages (specifically alarms). Therefore, a 50% probability of exceeding the threshold in publication messages will result in more than 50% of additional traffic in the cloud communication compared to the edge, even though exactly half of the packets are alarms. In order to capture the traffic in this experiment, the Wireshark network analyzer has been used.

## 6. Results and Discussion

**Figure 9:**
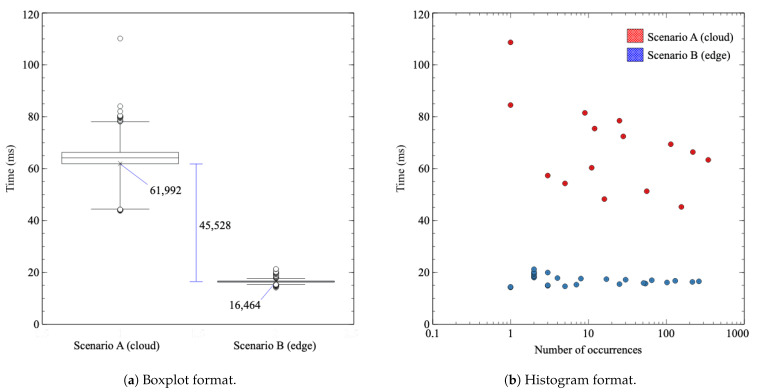
Latency measurement experiment for scenario A (**test 1.1,** regular cloud broker) and scenario B (**test 1.2**, alarm edge broker).

In this section, the results of previously presented experiments are shown and analyzed. Firstly, when it comes to the first experiment involving tests **1.1** and **1.2**, Figure 9a shows response time measurement results. The abscissa axis represents the two evaluated scenarios (scenario A in the left and scenario B in the right), while in the ordinate axis, the actual response time values are provided. The boxplot has been calculated with 98 percentile and shows the median (horizontal line), the mean (x), the box (top limit 75 percentile and bottom limit 25 percentile), “whiskers” (that contain the 98% of results), and outliers (circles, the 2% that is left of results). Figure 9b shows the same response time measurements in a histogram format.

As the figure shows, the cloud case has a mean latency of 61.992 ms, along with a noteworthy variability that can be seen due to a wide distribution of results. However, the edge broker case has a mean of 16.464 ms and high consistency in the majority of results, comprising the 98% of them in a 3 milliseconds margin.

**Figure 10:**
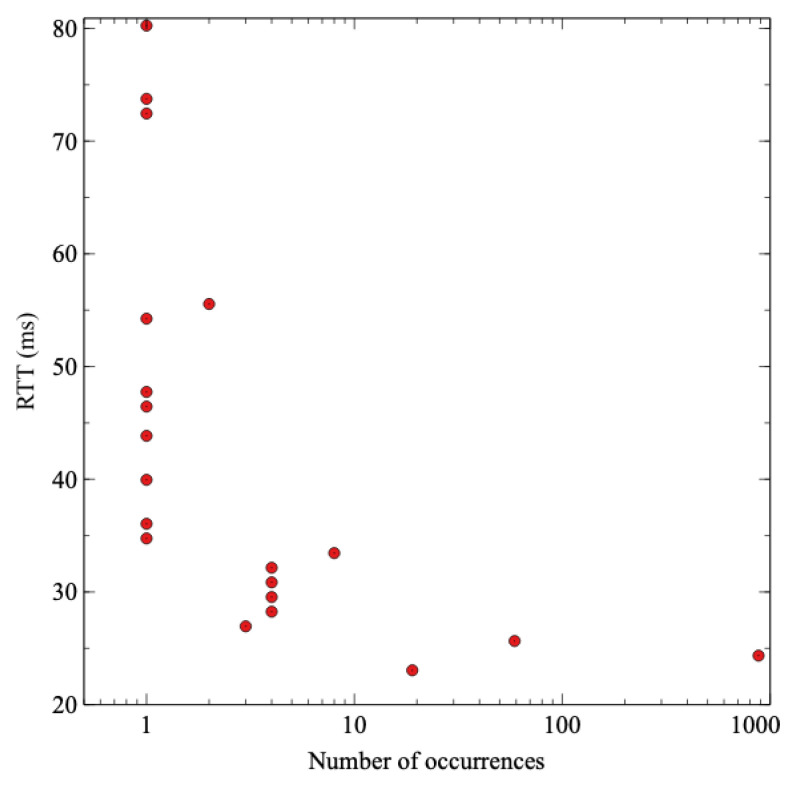
Histogram of latency measurements of ICMP Echo requests from sensor to the cloud broker.

The difference between both cases attends the expected behavior, being 74% lower in the case of scenario B (the edge broker and the INC with P4) than scenario A (the regular cloud communication). As it is logical, the communication with the cloud that crosses the ISP network is slower and more variable. Still, the difference also includes the extra processing that the cloud broker has to do in order to check if the sensor value exceeds the threshold. In the case of the edge broker, that processing is done in the SmartNIC with P4 code, but not for the cloud. To check the contribution of that extra processing, a ping has been performed against the cloud broker with the same packet length as MQTT publish messages to measure the RTT without that additional processing. Figure 10 shows the results of the latency taken with the ping tool from the publisher to the cloud broker. The mean time of the RTT is 25 ms, so it is safe to say that MQTT processing in the cloud broker introduces a delay of 40 ms. It is evident that additional processing in the cloud contributes to increasing the delay of the communication compared to the edge.

**Figure 11:**
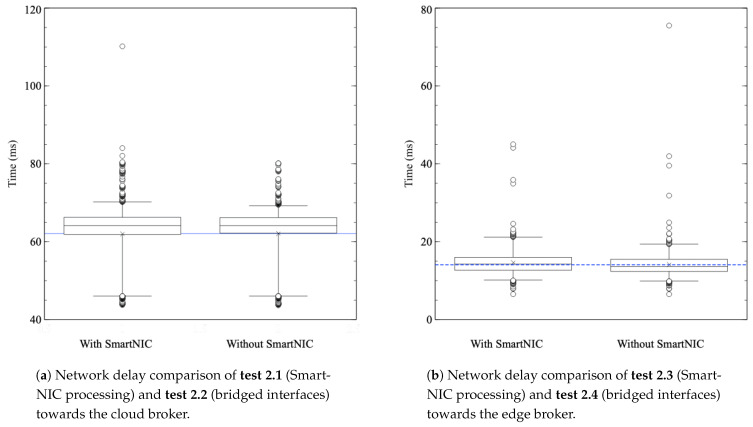
Network delay with and without SmartNIC processing. (**a**) Communications with the cloud broker. (**b**) Communications with the edge broker.

When it comes to the second experiment involving the processing delay introduced by the INC application, Figure 11a,b show the comparison between communications with and without the SmartNIC, towards the cloud broker (left-hand side, **tests 2.1 and 2.2**, in that order) and towards the edge broker (right-hand side, **tests 2.3 and 2.4**, in that order). When it comes to the first and second tests, Figure 11a, the disparity of both cases is almost indistinguishable. On the other hand, Figure 11b shows that direct processing has a slightly lower delay, a difference of only 0.501 ms between both cases (mean times of 14.607 ms with SmartNIC and 14.107 ms without SmartNIC). These results demonstrate that processing in the SmartNIC is efficient enough to prove its validity.

**Figure 12:**
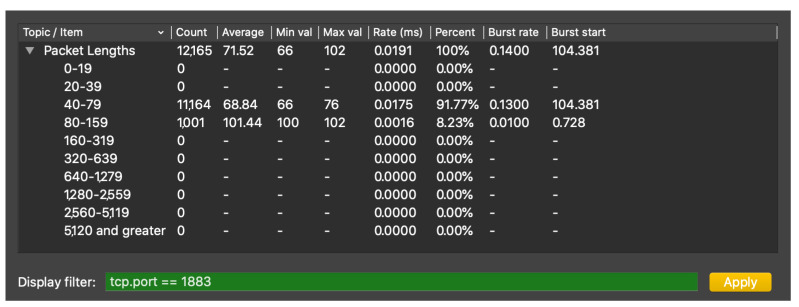
Screenshot of Wireshark packet length analysis of the experiment 3 MQTT communication.

Finally, throughput results are analyzed in the third experiment, using the case of 5% of values being generated above the threshold (**test 3.2**) to show the procedure of throughput calculation. In the case where the processing is performed at the cloud, as no preprocessing is performed at the SmartNIC, all the packets must be sent to the cloud in order to compare their conveyed value with the predefined threshold. With 5% of conveyed values being above the threshold, a total of 4082 MQTT packets have been sent, 1001 of which have “test” topic and 58 “alarm” topic (these are the alarm response messages generated by the cloud broker). The rest of MQTT packets are MQTT control packets, related to the connection establishment between the IIoT device and the broker. A total of 12,165 TCP packets were sent to the cloud, during a period of about 638 s. A capture of packet length statistics has been taken, which shows a total of 870,071 bytes sent and a mean throughput of 1363.75 bytes/s in that period. These statistics can be examined in the packet length analysis provided by Wireshark, as Figure 12 demonstrates.

In the case where MQTT packets are preprocessed at the SmartNIC and 5% of values are being generated above the threshold, only 71 MQTT packets have been sent to the edge broker. Fifty-one of those packets are publish messages with an “alarm” topic, while the rest are messages that maintain the connection (only for responses). Additionally, 135 TCP messages are related to the MQTT conversation, in a period of about 628 s. A total sum of 9459 bytes was sent, and the mean throughput in that period was 15.06 bytes/s.

**Figure 13:**
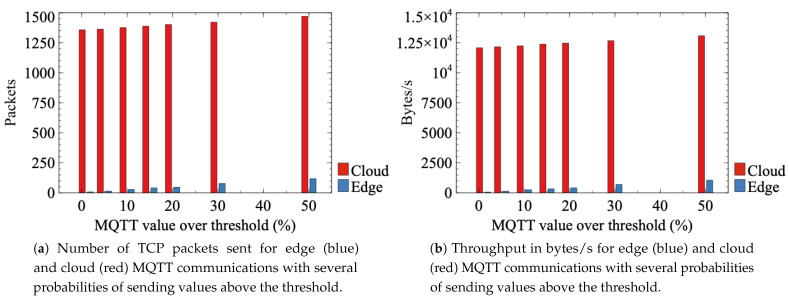
Comparison of throughput for edge and cloud MQTT communications with several threshold probabilities. (**a**) Number of TCP packets sent. (**b**) Throughput in bytes/s.

Figure 13a presents the total number of transmitted packets (TCP packets associated to MQTT conversations) and Figure 13b shows the equivalent throughput value. As shown in this figure, preprocessing at the SmartNIC allows reducing throughput by about 90%. This is due to the fact that, when packets are preprocessed at the SmartNIC, only those packets with values above threshold must be further transmitted to the edge broker. Additionally, the publication communications between the SmartNIC and the edge broker do not require all the TCP connection messages, which allows reducing the throughput even more in this case, since the majority of TCP control packets are avoided. On the contrary, if no preprocessing is performed at the SmartNIC, all the MQTT messages must be forwarded to the cloud so that they are processed there in order to check if the value they convey is above the threshold or not.

These results show that the packet processing system to detect high values is beneficial not only for response times, but for bandwidth usage reduction too. This resource usage decrease is a crucial point, due to the massive amount of sensors that can be deployed and their corresponding communications saturating the network. Besides, edge data processing centers are usually less powerful than cloud centers due to optimization of economic resources, which could result in a link or CPU saturation by the transmission of full sensor communication of a factory to the aforementioned edge broker.

## 7. Conclusions and Future Work

IoT technologies are growing in industrial environments due to their usefulness, from product manufacturing development to advanced monitoring in any process. Even though IoT is undoubtedly effective, there are still some challenges that must be overcome. Current services and applications have strict performance needs, such as low transmission delay or packet error rate, so network design and management must evolve in order to meet the requirements. Specifically, these parameters are extremely important for critical messages, such as threshold exceeding alarms in industrial environments. It should be said that, in a normal operation of an industrial process, there should be few situations in which a certain threshold is exceeded and these should be treated with minimal delay.

Therefore, our paper presents a solution that is based on the assumption that INC improves the efficiency of industrial processes thanks to a better network design. Our system proposes an architecture for the implementation of INC using DPP to provide response time and throughput enhancement in industrial environments, which follows the trend of current network development. Besides, our methodology implies experimental research using a real testbed to validate the concept and demonstrate the benefits of the proposed system. This is a crucial aspect of this paper, as it is fundamental to test and investigate the effective contribution of an idea to real environments. Results show that improved performance is achieved in terms of network parameters. In fact, DPP and P4 are demonstrated to be useful mechanisms for dealing with industrial communication challenges, and open the door for other possible applications.

Future works in this field may study other possible applications of the proposed solution. Apart from alarm generation, packets could be simply dropped if certain conditions are not met, therefore reducing bandwidth usage, improving energy savings and efficiency, and decreasing computing resource consumption. Additionally, the improvement of other performance parameters with data plane programming is also feasible. Research could be done in security through the implementation of advanced packet processing functions, or even the offloading of traditionally server side functions. Multiple protocols are resource-hungry for scarce resource devices, such as those that provide security related functions. These procedures could be implemented in specifically designed devices, using DPP to manage the communication among all concerned entities. Currently DPP is an open study field and there are a lot of applications that can be developed towards the enhancement of numerous network features. This, paired with industrial processes, can provide a suitable environment for research and innovation. Besides, factories are in the middle of the transition towards the implementation of 5G networks, which entails new challenges and opportunities. In this case, it is one of the main points of the new standard to bring computing resources closer to the final user, so similar approaches to the one proposed in this paper may be beneficial.

## Abbreviations

The following abbreviations are used in this manuscript:

IoT	Internet of Things
IIoT	Industrial Internet of Things
INC	In-Network Computing
DPP	Data Plane Programming
P4	Programming Protocol-Independent Packet Processors
MQTT	Message Queuing Telemetry Transport
IP	Internet Protocol
TCP	Transmission Control Protocol
XMPP	Extensible Messaging and Presence Protocol
OPC UA	OPC Unified Architecture, Object Linking and Embedding for Process Control (OPC)
CoAP	Constrained Application Protocol
UDP	User Datagram Protocol
SCTP	Stream Control Transmission Protocol
TLS	Transport Layer Security
ASIC	Application-Specific Integrated Circuit
NPU	Network Processing Unit
FPGA	Field-Programmable Gate Array
GPU	Graphical Processing Unit
CPU	Central Processing Unit
OVS	Open vSwitch
SN4I	Smart Networks 4 Industry
NFV	Network Function Virtualization
SDN	Software Defined Networking
MAC	Media Access Control
EIB	Escuela de Ingeniería de Bilbao
CFAA	Centro de Fabricación Aeronáutica Avanzada
WAN	Wide Area Network
ONOS	Open Network Operating System
OSM MANO	Open Source MANO, Management and network orchestration (MANO)
ISP	Internet Service Provider
KPI	Key Performance Indicator
RTT	Round Trip Time
